# Performance Analysis of Boosting Classifiers in Recognizing Activities of Daily Living

**DOI:** 10.3390/ijerph17031082

**Published:** 2020-02-08

**Authors:** Saifur Rahman, Muhammad Irfan, Mohsin Raza, Khawaja Moyeezullah Ghori, Shumayla Yaqoob, Muhammad Awais

**Affiliations:** 1 Electrical Engineering Department, College of Engineering, Najran University, Najran 61441, Saudi Arabia; miditta@nu.edu.sa; 2 Department of Computer and Information Sciences, Northumbria University, Newcastle-upon-Tyne NE1 8ST, UK; mohsinraza119@gmail.com; 3 Department of Computer Science, National University of Modern Languages, Islamabad 44000, Pakistan; mghouri@numl.edu.pk (K.M.G.); shumaylayaqoob@gmail.com (S.Y.); 4 Faculty of Medicine and Health, School of Psychology, University of Leeds, Leeds LS2 9JT, UK

**Keywords:** activities of daily living, boosting classifiers, machine learning, performance, physical activity classification

## Abstract

Physical activity is essential for physical and mental health, and its absence is highly associated with severe health conditions and disorders. Therefore, tracking activities of daily living can help promote quality of life. Wearable sensors in this regard can provide a reliable and economical means of tracking such activities, and such sensors are readily available in smartphones and watches. This study is the first of its kind to develop a wearable sensor-based physical activity classification system using a special class of supervised machine learning approaches called boosting algorithms. The study presents the performance analysis of several boosting algorithms (extreme gradient boosting—XGB, light gradient boosting machine—LGBM, gradient boosting—GB, cat boosting—CB and AdaBoost) in a fair and unbiased performance way using uniform dataset, feature set, feature selection method, performance metric and cross-validation techniques. The study utilizes the Smartphone-based dataset of thirty individuals. The results showed that the proposed method could accurately classify the activities of daily living with very high performance (above 90%). These findings suggest the strength of the proposed system in classifying activity of daily living using only the smartphone sensor’s data and can assist in reducing the physical inactivity patterns to promote a healthier lifestyle and wellbeing.

## 1. Introduction

World health organizations defined physical activity as any body movement that requires energy expenditure to perform any task originated through the musculoskeletal system [1]. Physical activity is quite essential for human beings to carry on their daily living routine work. Activities are the movements that the body does all day long. Picking up fruits, cleaning the house, sitting, standing, walking and lying are examples of activities of daily living (ADLs). Physical activity should not be confused with exercise since it is a sub-branch of physical activity. Exercise is a set of well-planned, structured and repetitive actions where we plan to move a specific part of the body, such as lower or upper limbs, and the movement is repeated, for example, lifting the weight ten times in a row. Similarly, brisk walking for at least 10 min is considered exercise [2]. Thus, exercise is an intentional effort to raise the heart rate, raise muscles and increase the flexibility of the body. However, both physical activity and exercise are essential in promoting a healthier lifestyle and wellbeing. Everyone needs exercise and activity each day. Therefore, incorporating ADLs and exercise in our daily routine can protect the human body from severe health conditions and diseases, such as cardiovascular disease, high risk of falling, dementia, obesity and depression [3].

Obesity is one of the leading medical issues in the modern world. The primary reason for obesity is an inactive lifestyle. Obesity has become a global epidemic, and the world health organizations are warning that one-third of the world population is now obese or overweight. Almost 10% of total medical costs in the USA are related to obesity issues [4,5]. Mainly, one in three adults or one in six children is obese in the USA. Obesity is one of the major causes of death in the USA [6,7]. It was reported in [8,9] that nearly 20,000 people die every year in Saudi Arabia due to obesity. Additionally, 36% of the Saudi population is obese, and almost 69% of Saudi people are overweight [10,11,12,13]. One of the main reasons for the significant number of obese in Saudi Arabia is its climate. The temperature remains high during day time, and people choose to drive cars rather than walking and doing physical activity. Obesity is linked with other diseases such as heart disease, diabetes, depression and dementia. Adopting a healthy lifestyle, selecting healthy food and having regular physical exercise and physical activity can reduce the risk of obesity [14].

The lack of physical activity may also cause dementia and Alzheimer’s disease [15,16]. The brain gets benefits from regular physical movement of the body. Scientists working on brain health have revealed a fantastic link between brain health and body activity. Daily activity triggers the growth of new blood vessels in the brain, allowing the brain to receive larger blood supply. It spurs the creation of new neurons in the brain’s memory centre. Studies in older adults have shown that regular aerobic exercise can improve cognitive function and slows the cognitive decline. Therefore, to maintain brain memory, peoples may consider starting and continuing a consistent healthy workout. Studies have shown that regular exercise can improve the mood and reduce the symptoms of anxiety, depression and dementia. It can even make you more resilient to stress [17,18,19].

## 2. Overview of Boosting Algorithms and Their Use in Physical Activity Classification Research

A recent development in the miniature sensing devices, considering their computational power, data storage capabilities, wear-ability and ease of use, made it possible to use them for activity monitoring. As a consequence, several activities tracking systems were developed using the accelerometer and gyroscope sensors to provide the acceleration and angular velocity measurements that can eventually assist in better quantification and profiling of ADLs. This section provides an overview of recent developments in the domain of physical activity classification (PAC) to classify ADLs using the boosting family of machine learning classifiers. It also provides the limitations of existing boosting-based PAC systems.

### 2.1. Boosting Algorithms

**Figure 1:**
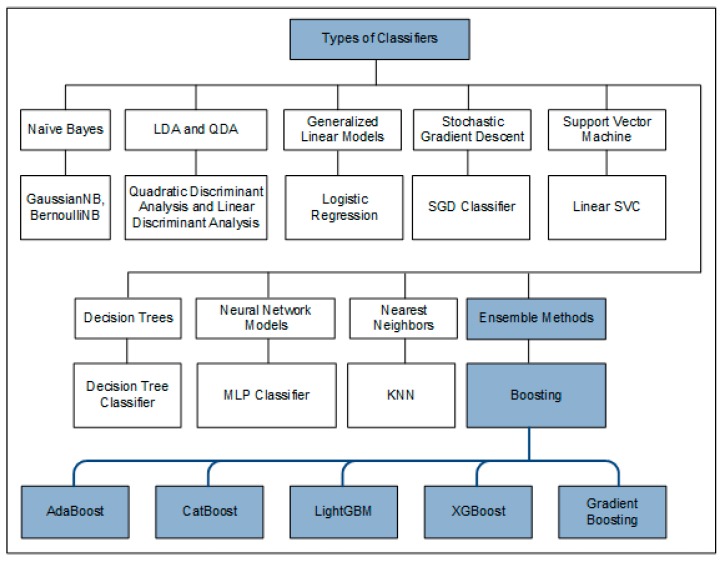
Basic types of supervised machine learning algorithms.

Supervised machine learning classifiers can be categorized into multiple types. These types include naïve Bayes, linear discriminant analysis (LDA) and quadratic discriminant analysis (QDA), generalized linear models, stochastic gradient descent, support vector machine (SVM), linear support vector classifier (Linear SVC) decision trees, neural network models, nearest neighbours and ensemble methods. The ensemble methods combine weak learners to create strong learners. The objective of these predictive models is to improve the overall accuracy rate. This can be achieved using two strategies. One of the strategies is the use of feature engineering, and the other strategy is the use of boosting algorithms. Boosting algorithms concentrate on those training observations which end up having misclassifications. There are five vastly used boosting methods, which include AdaBoost, CatBoost, LightGBM, XGBoost and gradient boosting. The basic hierarchy of supervised machine learning classifiers is shown in Figure 1.

#### 2.1.1. Adaboost

Adaptive boosting (AdaBoost) initially assigns equal weights to each training observation. It uses multiple weak models and assigns higher weights to those observations for which misclassification was observed. As it uses multiple weak models, combining the results of the decision boundaries achieved during multiple iterations, the accuracy of the misclassified observations is improved, and hence the accuracy of the overall iterations is also improved.

The weak models are evaluated using the error rate as given in (1):

(1) $$\varepsilon_{t}={\mathrm{Pr}}_{i∼D_{t}}\left[h_{t}\left(x_{i}\right)\neq y_{i}\right]=\sum_{i:h_{t}\left(x_{i}\right)\neq y_{i}}D_{t}\left(i\right)$$

where $\varepsilon_{t}$ is the weighted error estimate, ${\mathrm{Pr}}_{i∼D_{t}}$ is the probability of the random example i to the distribution $D_{t}$, $h_{t}$ are the hypotheses of the weak learner, $x_{i}$ is the training observation, $y_{i}$ is the target variable, t is the iteration number. The prediction error is one if the classification is wrong and 0 if the classification is correct.

#### 2.1.2. Gradient Boosting

Gradient boosting (GB) [20] sequentially creates new models from an ensemble of weak models with the idea that each new model can minimize the loss function. This loss function is measured by gradient descent method. With the use of the loss function, each new model fits more accurately with the observations, and thus the overall accuracy is improved. However, boosting needs to be eventually stopped; otherwise, the model will tend to overfit. The stopping criteria can be a threshold on the accuracy of predictions or a maximum number of models created.

#### 2.1.3. Lightgbm, Xgboost and Catboost

The structural difference between LightGBM [21] and XGBoost [22] is the way the best split is computed. For LightGBM, Gradient-based one-side sampling (GOSS) is used to identify the observations which can be used for computing the split. For XGBoost, a histogram-based algorithm filters the observations to be used for finding the split. The computation time of a histogram-based algorithm is more than the GOSS; therefore, in terms of complexity, LightGBM is generally more efficient as compared with XGBoost.

Another difference between them is the way each of the techniques handles categorical features. Boost [23] uses a one-hot encoding scheme to convert the categorical values into numerical values. It also enables users to supply the number of splits for a given categorical feature. An interesting observation about CatBoost is that it performs best when the dataset has the categorical features; otherwise, its performance is deteriorated on the absence of categorical features. A special algorithm [24] is used in LightGBM for the conversion of categorical values into numerical values. Contrary to CatBoost and LightGBM, XGBoost does not offer any conversion scheme for categorical features. All the categorical features must be converted into numerical features in the preprocessing step before the data is trained and tested for XGBoost. In general, XGBoost is slower as compared with LightGBM and CatBoost.

### 2.2. Use of Boosting Algorithms for PAC

The authors of [25] have used Adaboost as the base classifier for the recognition of five activities using a smartphone. The activities classified were walking, sitting, standing, cycling and running. Gyroscopes and acceleration sensors were used with the combination of smartphones for the collection of data. They have used decision stump as the subclassified in Adaboost, which is a one-layer decision tree that can classify based on a single feature of the observation. The authors conclude that they achieved 98% accuracy with this model. However, this work was limited to classifying only five activities. Similar work was performed in [26]. The authors have used decision trees and random forest in combination with Adaboost to classify five activities, namely, standing up, standing, sitting down, sitting and walking. They used decision tree and random forest classifiers as week learners and utilized a publically available human activity recognition dataset [27], which is developed by acceleration-based sensors placed on various body locations; (waist, right arm, left thigh and right ankle. The paper concluded that accuracies of 99.87% and 99.9% were achieved for decision tree and random forest, respectively. Precision and recall were the other performance evaluation metrics used. Reiss et al. [28] have compared multiple variants of Adaboost for multi-class classification in physical activity classification. Experiments were performed on eight different datasets from the repository of the University of California, Irvine (UCI) [29]. The paper included the classification of 21 different physical positions and reported an overall accuracy of 77.78%. The authors concluded that confidence-based variation of AdaBoost ConfAdaBoost.M1 outperformed the other variations of Adaboost in seven of the eight selected datasets.

Gradient boosting has also been tested in physical activity classification. In [30], the authors have experimented with gradient boosting and random forest in a dataset that contains data related to free-living conditions. The data was collected from 36 persons using smartphones. A total of 59 features were used in the dataset, while six positions were monitored, namely, standing, sitting, downstairs, upstairs, jogging and walking. The paper concluded that the overall accuracies of 99.03% and 99.22% were recorded for random forest and gradient boosting, respectively. Gradient boosting, Adaboost, random forest and decision tree were used in somewhat a related problem of identification of feet fidgeting [31]. This work has used accelerometers on shoes for the data collection of four positions of legs which are generally categorized as fidgeting. This study concluded that random forest has the highest accuracy among the other classifiers.

The authors of [32] have used wearable sensors to monitor running movements of 513 teenagers to compare the performances of SVM, decision tree, k-nearest neighbours (KNN), random forest and gradient boosted decision tree with the performance of their proposed optimized XGBoost model. Their model is based on the algorithm for the optimization of the Bayesian hyperparameter. The model classified the fitness level of each participant. The paper concluded that their proposed XGBoost model outperformed the other classifiers. In a recent contribution, Zhang et al. [33] have used barometer, gyroscope and accelerometer to record five movements of multi-floor indoor activities. The recorded movements were elevator taking, stair climbing, stillness, escalator taking and walking. The authors have compared the performance of XGBoost with the performances of random forest, KNN, SVM, multi-layer perceptron (MLP) and GBDT and claimed that XGBoost outperformed the other classifiers with an overall accuracy of 84.41% and an F-score of 84.19%. Gao et al. [34] have recently proposed a framework for human activity recognition that uses stack denoising autoencoder (SDAE) and LightGBM. The authors have used three datasets with different activity modes, which were classified into a static mode, dynamic mode and moving mode. They have compared the efficiency of their proposed framework with the efficiencies of CNN and XGBoost and claimed that their proposed framework outperformed the other classifiers with an overall accuracy of 95.99%. They also evaluated their framework with precision, recall and F-1 score. The literate survey is summarized in Table 1 to provide a better overview of recent advancements that happened in classifying ADLs by incorporating the boosting-based classifiers within the context of PAC.

### 2.3. Limitations In Existing Boosting-Based PAC Systems

In recent years, there has been active participation in the recognition of human activities. Interestingly, the research community has paid attention to applying the boosting techniques for the classification of physical activities. However, the performances of existing boosting classifiers based on PAC systems are incomparable with each other, and existing studies are unable to provide a better insight into which algorithms perform better than others due to the inconsistencies involved in their design process. The inconsistencies are as follows: different set of ADLs analysed (sitting, standing, running, jogging, etc.), data collected over different populations (young, elderly, healthy, unhealthy), difference in type of signals measured (acceleration signal, gyroscope signal, barometric pressure signal), different sensor locations (waist, wrist, ankle, etc.), diversities in the feature-set analysed (time, frequency, statistical descriptors, etc.), differences in cross-validation strategies for performance evaluation (10 fold, leave one subjects out, etc.) and use of different performance metrics (accuracy, f-measure, precision, recall, etc.). Although some studies in Table 1 are providing high accuracy, all these differences make their performance incomparable. For example, Guo et al. [32] used the XGboost classifier and achieved an f-measure score of 99%, while Zhang et al. [33] have achieved f-measure of only 84.14% using the same classifier. Moreover, the impact of feature selection methods on the performance of boosting classifiers is not studied systematically considering the domain of PAC, and very little is known about how these classifiers behave when the feature selection stage is incorporated before classification. To better address this issue, a benchmark analysis was carried out by Awais et al. [35], which provides the sequence of steps that can be performed to provide a balanced and unbiased performance analysis of different PAC systems using a different type of classifiers. This study also investigates the performance of recently developed Catboost classifiers for PAC. Therefore, the objectives of the present study are

1. To provide an insight into existing boosting-based PAC systems and to provide the limitations and weaknesses of these systems in providing a fair and unbiased analysis.
2. To provide a fair and unbiased performance comparison of boosting classifiers in profiling ADLs.
3. To study the impact of feature selection on the performance of boosting classifiers and to identify which classifiers perform better than others with and without feature selection approach.

## 3. Materials and Methods

### 3.1. Dataset

The dataset used in this study is a publically available dataset [36] developed using the waist-mounted smartphone. Thirty subjects participated in the data collection protocol, aged from 19 to 48 years. The activities of daily living (ADLs) performed by the subjects were sitting, standing, walking, lying, stairs up and stairs down. The sampling frequency of accelerometer and gyroscope data collected through the smartphone was 50 Hz. More details of the dataset are presented in Table 2. Each column in Table 2 (except the percentage column) refers to the total number of window instances, and each window instance contains 2.56 s of data recording. For example, walking activity contains 1722 window instances in the total dataset, and each window corresponds to 2.56 s.

### 3.2. Feature Set

The feature set used to develop the PAC system is the same as reported in [36]. The acceleration signals were decomposed into gravitational and body acceleration components using the Butterworth filter of 3rd order. The angular speed and angular acceleration signals also resulted in five signals obtained through accelerometer and gyroscope. The magnitude signals of these five signals were then derived, resulting in a total of ten signals. The frequency components of these signals were also computed to better recognize the ADLs by exploring both the time and the frequency domain features.

Several features were extracted from these time and frequency signals across a window of 2.56 s, resulting in 128 samples per window with an overlap of 50%. The features from the aforementioned derived signals were mean, standard deviation, median, maximum, minimum, signal magnitude area, signal energy, interquartile range, entropy, autoregression coefficients, correlation coefficients, skewness, kurtosis, maximum frequency component and the angle between two vectors [36]. A total of 561 features were extracted from the window of 2.56 s.

### 3.3. Feature Selection

We incorporated feature selection before the classification stage to get rid of correlated features, as these have significant implications on the system performance and the computational complexity of the system considering the real-time applications. Correlation-based features (CFS) were used in this study to get rid of the redundant features. It is a statistical approach and provides a correlation score, which can then infer how much linear dependency exists between two features. The higher the correlation score between two features, the more linearly dependent the feature are with each other. Similarly, the lower the correlation score is, the less dependent the features are with each other. Therefore, low correlation features are eventually retained in the feature set, and highly correlated features are dropped from the feature set to reduce the redundancy and dependency among the features. In this paper, the correlation score of 0.8 was used as a threshold, and features above this threshold were eliminated from the feature set.

### 3.4. Classification and Cross-Validation

A total of five boosting-based machine learning classifiers were used in this study to observe their performances in classifying the ADLs. The classifiers are extreme gradient boosting (XGB), light gradient boosting machine (LGBM), gradient boosting (GB), cat boosting (CB) and AdaBoost. Two variants of AdaBoost were used, one using decision trees (ADA-DT) as a week learner and the other using random forest (ADA-RF) as a week learner. The classifiers’ settings were maximum depth = 50, minimum child weight = 1, number of estimators = 100, learning rate = 0.16 for XGB; maximum depth = 50, learning rate = 0.1, number of estimators = 100 for LGBM; learning rate = 0.15, depth = 10, loss function = multi class for CB; maximum depth = 10, number of estimators = 100 for ADA-DT; and maximum depth = 100, number of estimators = 100 for ADA-RF.

The dataset was divided into two datasets (70%/30%, training/testing) to avoid any bias in training and testing. Of the data, 70% was used to train the ML model, and the remaining 30% was used for testing the performance of the proposed activity classification system.

The expressions to calculate precision and recall are provided in Equations (2) and (3). Precision provides a measure of how accurate your model is in predicting the actual positives out of the total positives predicted by your system. Recall provides the number of actual positives captured by our model by classifying these as true positive.

F-measure can provide a balance between precision and recall, and it is preferred over accuracy where data is unbalanced. Therefore, F-measure was utilized in this study as a performance metric to provide a balanced and fair measure using the formula in (4).

(2) $$Precision=\frac{TP}{TP+FP}\times 100$$

(3) $$Recall=\frac{TP}{TP+FN}\times 100$$

(4) $$F-measure=2*\frac{Precision*Recall}{Precision+Recall}\times 100$$

where *TP*—True Positive, *FP*—False Positive, *FN*—False Negative.

## 4. Result and Discussion

### 4.1. Overall Performance Analysis of Boosting Classifiers Used With and Without Feature Selection

#### 4.1.1. Using All Feature Set

The performances of the six classifiers implemented in this study were computed using the F-measure metric. The performance of each classifier was computed using both feature sets, without feature selection, by using all the feature set obtained originally (All feat), and with feature selection, by only utilizing the uncorrelated feature set obtained through CFS approach.

**Figure 2:**
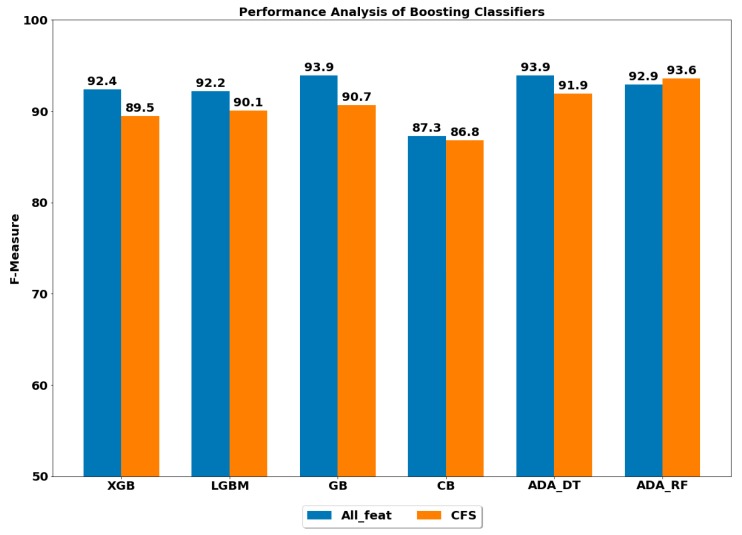
Performance analysis of various boosting classifiers with and without feature selection.

The overall performances of six classification algorithms to classify ADLs are depicted in Figure 2. The best overall performance of 93.9% was achieved by GB and ADA (DT) classifiers, and the lowest performance of 87.3% was obtained by the CB classifier using all the feature set. Moreover, the XGB, LGBM and ADA (RF) classifiers also performed significantly well by achieving the overall performance of above 90%, and the differences in performance of these classifiers are quite small (less than 1%). The confusion matrix of the best classifier, i.e., GB, using all feature set is presented in Table 3.

#### 4.1.2. Using Reduced Feature Set

The performance analysis of classification algorithms used in the study suggested that CFS-based feature set had decreased overall performance of the majority of the classifiers, except for ADA (RF), where the performance was increased. Although the change (increase or decrease) in the performance was not quite significant as compared with the performances achieved through the whole feature set, the number of features was significantly reduced from 561 to 150 using the CFS approach (over 70% reduction in several features). The number of features has implications on the computational complexity of the system [35,37]. A large number of features increases the computational complexity of the system and makes the systems infeasible to operate in real-time scenarios. On the contrary, the reduced feature set can significantly reduce the computational overheads and computational complexity of the system, which can be implemented in real-time applications. The confusion matrix of best-performing classifiers, all using the reduced CFS feature set, is presented in Table 4.

### 4.2. Performance Analysis of Individual ADL Classified by Boosting Classifiers

**Figure 3:**
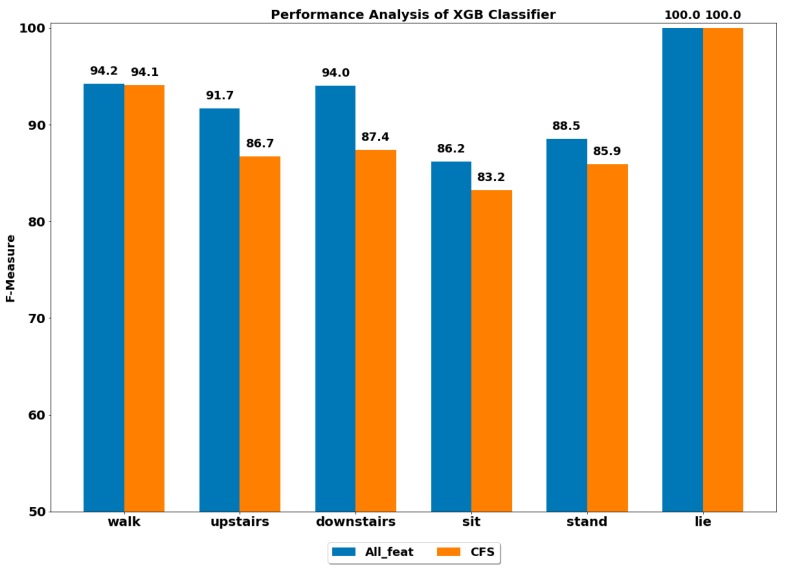
Performance analysis of XGB (extreme gradient boosting) classifier with and without feature selection to classify set of ADLs (activities of daily living).

**Figure 4:**
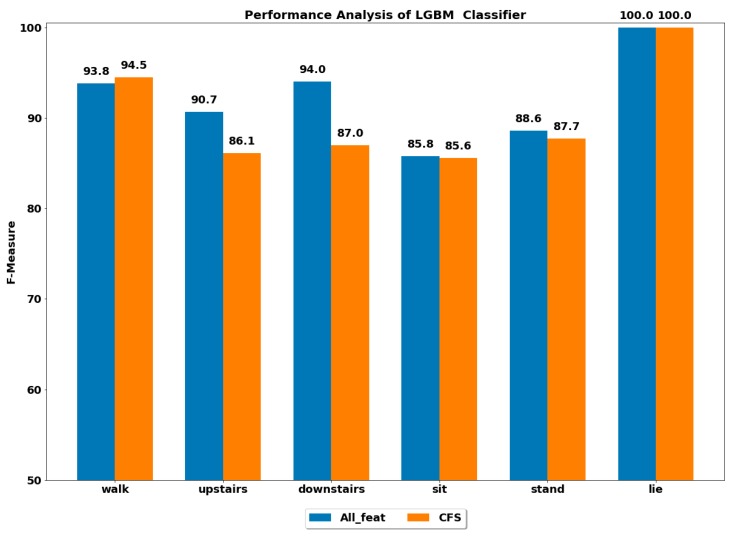
Performance analysis of LGBM (light gradient boosting Machine) classifier with and without feature selection to classify set of ADLs.

**Figure 5:**
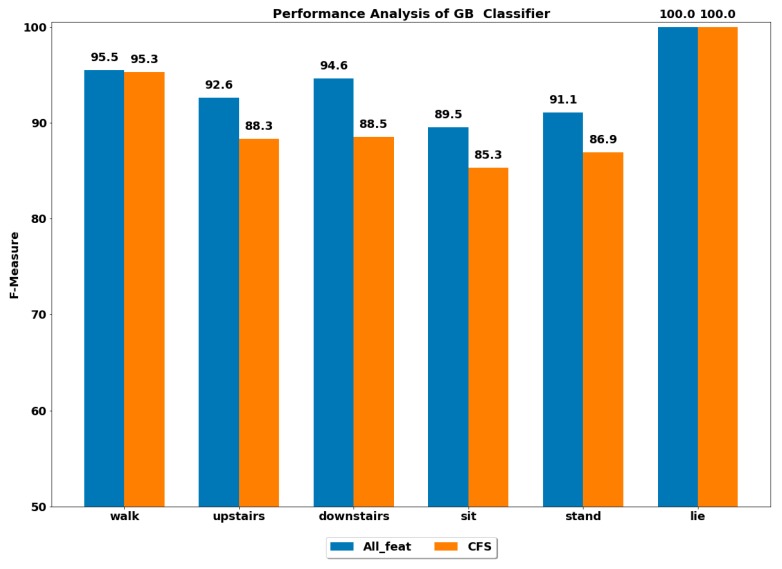
Performance analysis of GB (gradient boosting) classifier with and without feature selection to classify set of ADLs.

**Figure 6:**
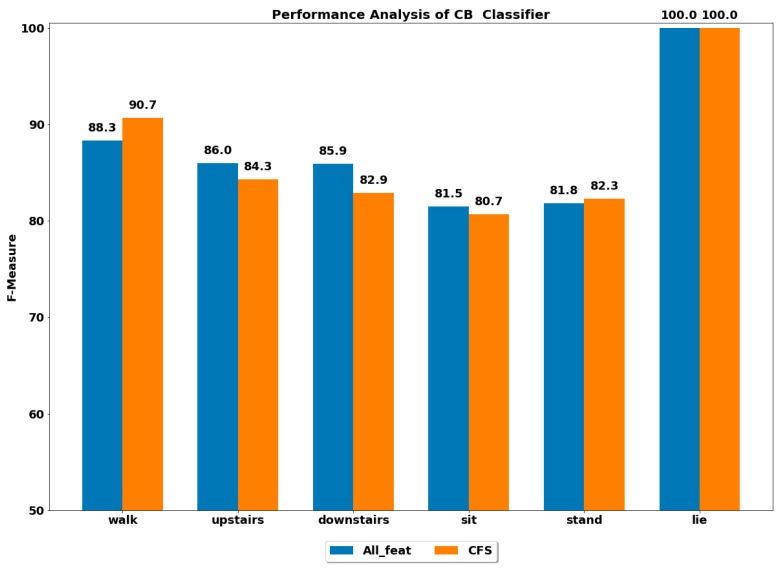
Performance analysis of CB (cat boosting) classifier with and without feature selection to classify set of ADLs.

**Figure 7:**
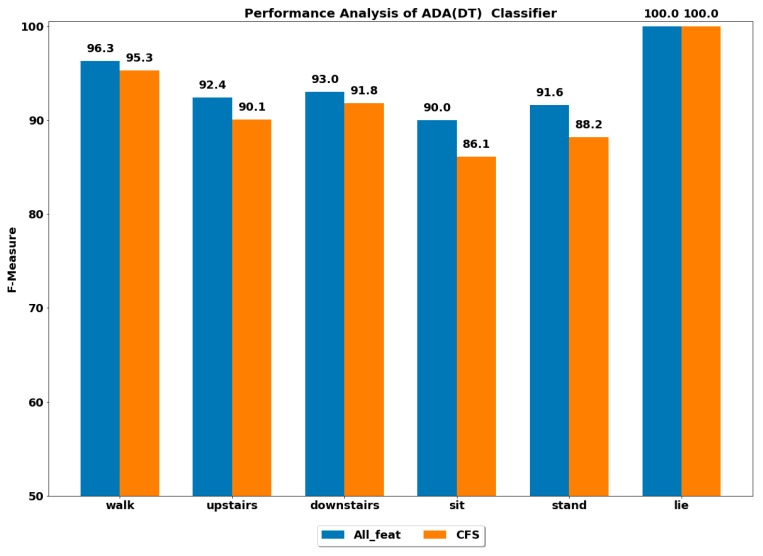
Performance analysis of ADA (DT) classifier with and without feature selection to classify set of ADLs.

**Figure 8:**
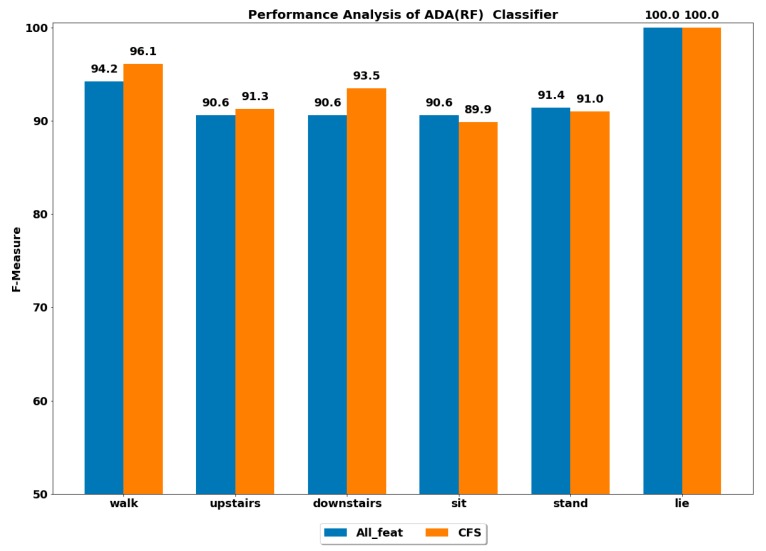
Performance analysis of ADA (RF) classifier with and without feature selection to classify set of ADLs.

The performance of all the boosting classifiers was also computed to classify each ADL using all feature set and using only CFS-based reduced feature set. The performances by each class of ADL for the classifiers XGB, LGBM, GB, CB, ADA (DT) and ADA (RF) are depicted in Figure 3, Figure 4, Figure 5, Figure 6, Figure 7 and Figure 8.

#### 4.2.1. Using All Feature Set

The best performance of above 96% was achieved by the ADA boost classifier using all feature set to classify the walking activity, while the CB classifier performed the worst among all. All the gradient boosting classifiers’ performances were quite close to that of ADA boost classifier with an overall performance of above 92% in classifying the walking activity.

The GB classifier achieved the best performances of 92.55% and 94.61% in classifying the stairs up and stairs down activities, respectively. The other classifiers also performed well in classifying stairs up and stairs down with F-measure of above 90%, except for CB, whose performance was lowest among all.

The sedentary ADLs, i.e., sitting, standing and lying, were best classified by ADA boost classifiers using the all feature set, followed by the GB classifier. The performance of above 90% was achieved in classifying the sitting and standing by the ADA boost classifiers. The other classifiers’ performances were below 90%. All the classifiers accurately classified the lying class with excellent performance of 100%, including the CB classifier, whose performance was quite low in classifying the rest of the ADLs. This could be because the orientation of accelerometer signals was significantly changed during the lying stage when compared with other classes, thus helping in classifying lying accurately.

#### 4.2.2. Using Reduced Feature Set

The performance by a class of each ADL was computed on the reduced feature set using all the classifiers. The best performance by class was achieved using the ADA boost classifiers, while the next best performer was GB classifier.

The ADA (RF) outperformed all the classifiers in classifying all the ADLs, i.e., walking, stairs up, stairs down, sit, stand and lie.

These findings show the potential of using the reduced set of features to achieve the same performance and can potentially reduce the computational complexity of the PAC system by up to 70%.

### 4.3. Smartphone-Based Activity Profiling

The performances achieved to classify the ADLs through the GB classifier (using all the feature set) and ADA (RF) classifier (using reduced feature set) are quite encouraging and suggest that smartphone-based inertial sensors can provide a reliable measure to profile the ADLs. Smartphones are easy to carry in daily life routine and do not create many issues regarding battery life, since it is quite a routine matter to recharge the battery every day. Surprisingly, as an advantage, one can keep track of each of the ADLs performed along the course of a day, to weeks, months and even years. In this manner, one can adopt a heather lifestyle by incorporating more activity in their daily routines. Activity profiling can also provide an accurate and reliable measure to healthcare infrastructure and staff to provide well-informed interventions and therapies that can help the general population in obtaining a better quality of life and wellbeing.

## 5. Conclusions

This study provides a fair and unbiased performance analysis of the boosting classifiers used for physical activity classification. The study also investigates the performance of newly developed CatBoost classifiers and how well they performs with and without feature selection scenarios. The fair and unbiased performance analysis is accomplished by keeping the uniformity across the studied population, set of ADLs performed, the sensor type, the sensor location, the feature set, the cross-validation procedure and the performance metric. Gradient boosting classifier performed best among all classifiers when analysed over the whole feature set, and the achieved performance was around 94%. ADA boost classifier using the random forest as weak learner achieved the same level of performance. However, the ADA (RF) used a significantly reduced feature set (over 70% reduction) as compared with GB classifier while achieved the same performance in classifying ADLs. The reduced feature set has implications in real-time implementation as this is directly linked with the computational complexity of the system. The large feature set can increase the computational complexity of the system, making it difficult for real-time implementations, while a reduced feature set can potentially improve the computation complexity of the system, making real-time implementation more feasible.

The study also come up with limitations. The main limitation is the context where ADLs are performed. This dataset was developed in a relatively controlled environment, and it would be interesting to see how the boosting-based classifiers perform when implemented on a dataset collected in free-living conditions without any constraint on where and how the activities are performed.

## Figures and Tables

**Table 1:** Overview of the physical activity classification systems developed using boosting classifiers.

Author	Sensor	Activities	Classifiers	Metrics	Result
Li et al. [25]	Gyroscopes, acceleration sensors	Walking, sitting, standing, cycling and running	Adaboost (base classifier), Decision Tree (weak learner)	Accuracy	98%
Zubair et al. [26]	Accelerometer	Standing up, standing, sitting down, sitting and walking	Adaboost (Decision Tree, Random Forest)	Accuracy	99.9% Accuracy of Adaboost
Reiss et al. [28]	Accelerometer	Descending and ascending stairs, walking, cycling, running, standing, sitting and laying	Adaboost	Accuracy	77.78%
Lee et al. [30]	Smartphones	Standing, sitting, downstairs, upstairs, jogging and walking	Gradient boosting, Random Forests	Accuracy	99.03% 99.22%
Esseiva et al. [31]	Accelerometers	Four positions of the leg for feet fidgeting; upper leg swinging, up and down leg bouncing, lower leg swinging, foot jiggling	Gradient boosting, Adaboost, random forest and decision tree	Accuracy, precision, recall, F-score	95% accuracy for gradient boosting
Guo et al. [32]	Smart bands	Four levels of fitness; excellent, good, medium, poor	XGBoost,	F-measure	99% for XGBoost
Zhang et al. [33]	Barometer, gyroscope and accelerometer	Elevator taking, stair climbing, stillness, escalator taking and walking	XGBoost,	F-measure	84.19% for XGBoost
Gao et al. [34]	Accelerometer, gyroscope, magnetic and pressure sensor	Static mode, dynamic mode and moving mode	SDAE with LightGBM	Accuracy	95.99%

**Table 2:** Overview of the physical activity classification systems developed using boosting classifiers.

Activity Type	Total Dataset	Percentage (Total Dataset)	Train Split	Test Split
Walk	1722	16.72%	1226	496
Upstairs	1544	14.99%	1073	471
Downstairs	1406	13.65%	986	420
Sit	1777	17.25%	1286	491
Stand	1906	18.51%	1374	532
Lie	1944	18.88%	1407	537

**Table 3:** Confusion matrix of best-performing classifier (GB) using all feature set.

	Predicted	Walk	Upstairs	Downstairs	Sit	Stand	Lie
Actual							
**Walk**		486	6	4	0	0	0
**Upstairs**		29	435	6	1	0	0
**Downstairs**		7	26	386	0	1	0
**Sit**		0	2	0	424	65	0
**Stand**		0	0	0	32	500	0
**Lie**		0	0	0	0	0	537

**Table 4:** Confusion matrix of best-performing classifier ADA(RF) using reduced feature set.

	Predicted	Walk	Up-Stairs	Down-Stairs	Sit	Stand	Lie
Actual							
**Walk**		491	1	4	0	0	0
**Upstairs**		33	428	10	0	0	0
**Downstairs**		2	37	381	0	0	0
**Sit**		0	1	0	436	54	0
**Stand**		0	0	0	43	489	0
**Lie**		0	0	0	0	0	537
